# Effects of portable pedal machines at work on lipoprotein subfraction profile in sedentary workers – the REMOVE study

**DOI:** 10.1186/s12944-024-02098-w

**Published:** 2024-04-14

**Authors:** Hijrah Nasir, Frederic Dutheil, Ines Ramos, Terry Guirado, Sarah de Saint-Vincent, David Thivel, Lore Metz, Martine Duclos

**Affiliations:** 1 Laboratory of the Metabolic Adaptations to Exercise under Physiological and Pathological Conditions (AME2P), Université Clermont Auvergne, Clermont-Ferrand, France; 2 CNRS, LaPSCo, CHU Clermont-Ferrand, Occupational and Environmental Medicine, Université Clermont Auvergne, Clermont-Ferrand, France; 3 CHU Clermont-Ferrand, DRCI, Biostatistics, Clermont-Ferrand, France; 4 Laboratory of the Metabolic Adaptations to Exercise under Physiological and Pathological Conditions (AME2P), CRNH, Université Clermont Auvergne, Clermont-Ferrand, France; 5 Institut de Médecine du Travail, Université Clermont Auvergne, Clermont-Ferrand, France; 6 INRAE, UNH, CHU Clermont-Ferrand, Department of Sport Medicine and Functional Exploration, Université Clermont Auvergne, Clermont-Ferrand, France

**Keywords:** Lipoproteins, Cardiovascular risk, Physical activity, Sitting, Occupation

## Abstract

**Background:**

Sedentary behaviour at work is a major cause of atherosclerosis, particularly in tertiary workers. However, no studies have ever assessed the effect of active workstation on lipoprotein subfraction profile. This study aimed to evaluate the effect of 12-week portable pedal machines (PPMs) on lipoprotein subfraction profile among healthy sedentary workers.

**Methods:**

Healthy administrative workers were randomized into an intervention group using PPMs for 12 weeks or a control group using normal-desk. Lipoprotein subfractions were assessed using Lipoprint® electrophoresis. Main outcomes were explored using mixed models with sensitivity analyses (four models).

**Results:**

We included 40 participants (43.7 ± 8.6 years old, 100% women, BMI 23.8 ± 3.4 kg/m^2^; sedentary time at work 7.7 ± 1.8 h/day). Groups did not differ at baseline in any outcomes. 32 participants finished the trial. Changes in lipoprotein subfractions were especially marked for LDL profile. There was an interaction time x group for all parameters related to LDL and their subfractions: total LDL-cholesterol (*p* = 0.012), LDL particle size (*p* = 0.027), large LDL subfractions 1 and 2 (*p* = 0.001), and small dense LDL subfractions 3 to 7 (*p* = 0.046), using the crude model. The interaction reflects difference in the direction of changes between groups. The LDL particle size significantly increased in the intervention group (from 271.9 ± 2.5 at t0 to 272.8 ± 1.9 Ångström at t1, *p* = 0.037) while it did not change in the control group (272.5 ± 1.7 at t0 to 271.8 ± 1.5Å at t1, *p* = 0.52). All interactions were constantly significant whatever the models. Influencing variables were mainly stress at work that was associated with an increase in total LDL-cholesterol (coefficient 3.15, 95CI 0.20 to 6.11 mg/dl, *p* = 0.038), and BMI that was associated with Large-LDL, Large-HDL, IDL-C and triglycerides.

**Conclusions:**

Lipoprotein profile was improved after a 12-week PPMs intervention at work in healthy administrative workers. Changes were mainly showed for LDL and LDL subfractions. Lipoprotein profile was worsened by stress at work, BMI and age.

**Trial registration:**

NCT04153214.

**Supplementary Information:**

The online version contains supplementary material available at 10.1186/s12944-024-02098-w.

## Introduction

Cardiovascular disease is a public health issue [1, 2], with atherogenicity being responsible to most of cardiovascular events [3]. The two main historical factors of atherogenicity are a reduced high-density lipoprotein cholesterol (HDL) and increased low-density lipoprotein cholesterol (LDL) [4]. Very interestingly, historical research showed that LDL and HDL are composed of several subtractions of lipoproteins, with only the smallest and dense subfractions being atherogenic [5]. Using electrophoresis, HDL can be categorized into small, intermediate, and large subfractions [6], and LDL can be categorized into seven subfractions, numbered from 1 to 7 (from the largest to the smallest) [7]. LDL subfractions 1 and 2 are large and buoyant, while LDL subfractions 3 to 7 are small and dense. Small dense LDL (sdLDL) has been associated with insulin resistance and increased risk of cardiovascular disease [8, 9]. SdLDL has a low affinity for LDL receptors and a low catabolic rate, thus remaining longer in the circulation [10]. They increase susceptibility to oxidative stress and can penetrate easily into the arterial wall due to their small diameter [11]. SdLDL is a strong independent predictor of coronary heart disease [10, 12]. Sedentary behaviour is one of the primary causes of cardiovascular disease [13, 14] and a growing public health issue [15]. Prolonged sitting time was associated with poorer health-related outcomes linked with the atherosclerotic process such as levels of lipoproteins – total, HDL and LDL cholesterol [16, 17]. In developed countries, sedentary behaviour is a major cause of preventable mortality with many workers spending a third of their waking time sitting at work [18]. Therefore, sedentary behaviour must be a major target of workplace preventive strategy, especially for workers from tertiary sector [19]. To break sedentary behaviour, light physical activity at work have been promoted using active workstation, such as treadmills, cycling desk, or portable pedal machines (PPMs) [20]. Among active workstation, PPMs are the easiest to implement at work [20]. The efficiency of active workstations to increase physical activity at work in overweight and obese individuals has been tested in several studies, but few studies assessed their effect on normal weight individuals [21]. However, to our knowledge, there is no study that assessed the effect of active workstation on lipoprotein subfractions.

Several variables such as age, sex, body mass index (BMI), moderate-to-vigorous physical activity (MVPA), stress, and sleep quality are long reported as influencing factors of lipoprotein profile and must be controlled when searching for an effect of active workstation on the atherosclerotic risk. Increasing age was found to be positively correlated with sdLDL [22]. In terms of sex, sedentary behaviour has been found to have a more deleterious effect on lipoprotein profile in women [23]. Higher BMI was linked with worst lipoprotein profile [7]. Besides breaking sedentary behaviour by active workstation at work, regular MVPA is also important to improve the lipoprotein subfraction profile [5, 10, 24]. In addition, stress at work was also a main factor linked to cardiovascular disease [25, 26], and possibly through changes in lipoprotein subfraction profile [27, 28]. Lastly, sleep duration was longitudinally and significantly associated with a poorer lipid profile [29].

Therefore, we aimed to investigate the effect of a 12-week PPMs intervention on lipoprotein subfractions among healthy inactive office workers. Secondary objectives were to assess the effect of traditional influencing variables of lipoprotein profile such as sociodemographic, BMI, MVPA, stress, and sleep quality.

## Methods

### Study design

This study was a randomised controlled trial. Directors of administrative companies who agreed on the participation of their staff, informed their workers about the study. Volunteers meeting eligibility criteria were randomly allocated in a control group or an intervention group. All participants provided their written informed consent. This study received approval from the French ethical committee (Comité de Protection des Personnes Ile-de-France VIII) and was conducted according to the declaration of Helsinki. The study has been registered at ClinicalTrials.gov (NCT04153214).

### Participants

Inclusion criteria were: physically inactive (less than 150 min of moderate-to-vigorous physical activity per week), BMI ≤ 25 kg/m^2^, stable body weight or < 3 kg change over the previous 6 months, living a sedentary lifestyle (75% of working time spent in sitting position), not pregnant or lactating, no cardiovascular or metabolic disorders, no restricted diet during at least 6 months, no use of medications except oral contraceptive, and able to use and complete the PPM at work and/or during teleworking. Exclusion criteria were the occurrence of any medical issues during the study, such as any endocrinological disease or treatment that could interfere with levels of lipoprotein subfractions. Orthopaedic issues preventing the use of PPMs were also an exclusion criterion.

### Intervention

During the study, the intervention group was asked to cycle on a PPM (DeskCycle; 3D Innovations LLC, Greeley, CO) installed and adjusted with their seat desk. Participants were asked to perform PPM for 60 min per working day for 12 weeks, continuously or fractionated. The level of resistance for all the pedal was set on the minimal magnetic resistance (between 16 and 25 Watts). All participants reported their distance and the amount of cycled time every day that was shown by PPM monitor. This information was collected in weekly basis by the research team. The control group was asked to maintain their usual working habits, on their usual desk. Participants were asked to maintain their habitual daily physical behaviour and diet during the whole duration of the study. Diet was also controlled by self-reported questionnaires.

### Measurement time

All the data were collected twice: at baseline and at the end of the 12-week intervention.

#### Main outcome

Lipoprint® electrophoresis (Quantimetrix Inc., Redondo Beach, California) was used to assess the profile of lipoprotein subfractions. Fasting serum or plasma was analysed using this device to measure lipoprotein fractions and subfractions from VLDL to HDL. The procedure was started by loading and stacking gels. After the separating gel-matrix, lipoprotein particles move to different band based on their particle sizes. In this stage, HDL moves to the farthest followed by sdLDL, larger-buoyant LDL, Mid-bands (comprising primarily IDL) and VLDL. Before the test, all participants were asked to have a 12-hour fasting prior to blood sampling, as well as to avoid drinking coffee or tea at least 24 h before, keep their habitual activities, and to avoid stress. A nurse collected blood samples (10 mL) by veinous puncture. The blood samples were centrifuged, aliquoted, and stored at -80 °C until analysis. Total, HDL- and LDL-cholesterol, as well as triglycerides were assessed in the biochemistry laboratory of CHU Clermont-Ferrand, France; and the profile of lipoprotein subfractions using Lipoprint® electrophoresis were analysed in the laboratory of Occupational medicine from Université Clermont Auvergne, by a technician specialized in Lipoprint® electrophoresis.

#### Secondary outcomes

The measurement included in the study consisted of sociodemographic parameters (age, sex), body weight and height to calculate BMI, MVPA and sedentary behaviour, and levels of stress at work and at home, as well as sleep quality.

MVPA was assessed using ActiGraph wGT3X-BT accelerometer (Actigraph® Inc, Pensacola, FL), and sedentary behaviour was assessed using the ActiGraph conjugated with the activPAL3 inclinometer [30, 31]. Participants wore these devices during 7 consecutive days, at baseline as well as at the end of the intervention – thus including workdays and weekend. During the two periods of 7 days of measure, the ActiGraph was placed at the right hip using an elastic belt during waking hours and was removed at night, while the activPAL3 was continuously attached to the anterior midline of the right thigh using a nitrile sleeve, including at night. However, data were only analyzed when participant wore both devices. The Actigraph data were converted from frequency of 60 Hz into counts/min, using the ActiLife software (v6.13.4). MVPA was defined as > 2690 counts/min. The activPAL3 data were converted from 15-second epochs at a frequency of 20 Hz into time spent sitting/lying or standing/walking, using the activPAL software (v8.11.6.94). Sedentary behaviour was defined as time spent sitting/lying (activPAL) without being active i.e. <150 counts/min (Actigraph).

Stress at work, stress at home, and sleep quality were measured by self-reported questionnaires using visual analogue scales i.e. non-calibrated horizontal lines ranging from 0 (no stress / very bad sleep) to 10 (maximal stress / very good sleep) [32].

### Statistical analysis

The statistical analyses were carried out with the R software (version 4.3.1, R Core Team, Vienna, Austria), considering a risk of two-sided first type error of 5%. Quantitative variables were described by their mean ± standard deviation (SD), and categorical variables were presented as number and associated percentage (%). The normality of the data was analysed graphically and by a Shapiro-Wilk test. The quantitative variables were compared between the groups (intervention vs. control) at t0 and t1 by a Student t-test or a Mann-Whitney test when the conditions for applying the t-test were not respected. A delta was also calculated (Δ = value at t1 – value at t0) and compared between the 2 groups using a Student or Mann-Whitney test if applicable. The values of lipoproteins were also compared between t0 and t1 in each group by a paired Student t-test or a paired Wilcoxon test if the t-test was not applicable. The time effect (t1 vs. t0), group effect (intervention vs. control) and the interaction of both on the values of lipoprotein subfractions were assessed using a mixed linear regression model, with time, group and interaction factors as co-variables and the individual factor as a random effect. Sensitivity analyses were conducted, and a multivariate approach was used to adjust the previous models on other clinically relevant factors, such as age, BMI, MVPA, sedentary behaviour, stress at work and stress at home, as well as sleep quality. Sensitivity analysis included 4 model. Model 1 was a crude model; model 2 was the crude model 1 plus age and BMI; Model 3 was model 2 plus MVPA and sedentary time; and model 4 was model 3 plus stress at work, stress at home, and sleep quality. The results were presented as coefficient, 95% confidence intervals (95CI) and corresponding *p*-values.

## Results

### Participants

**Fig. 1:**
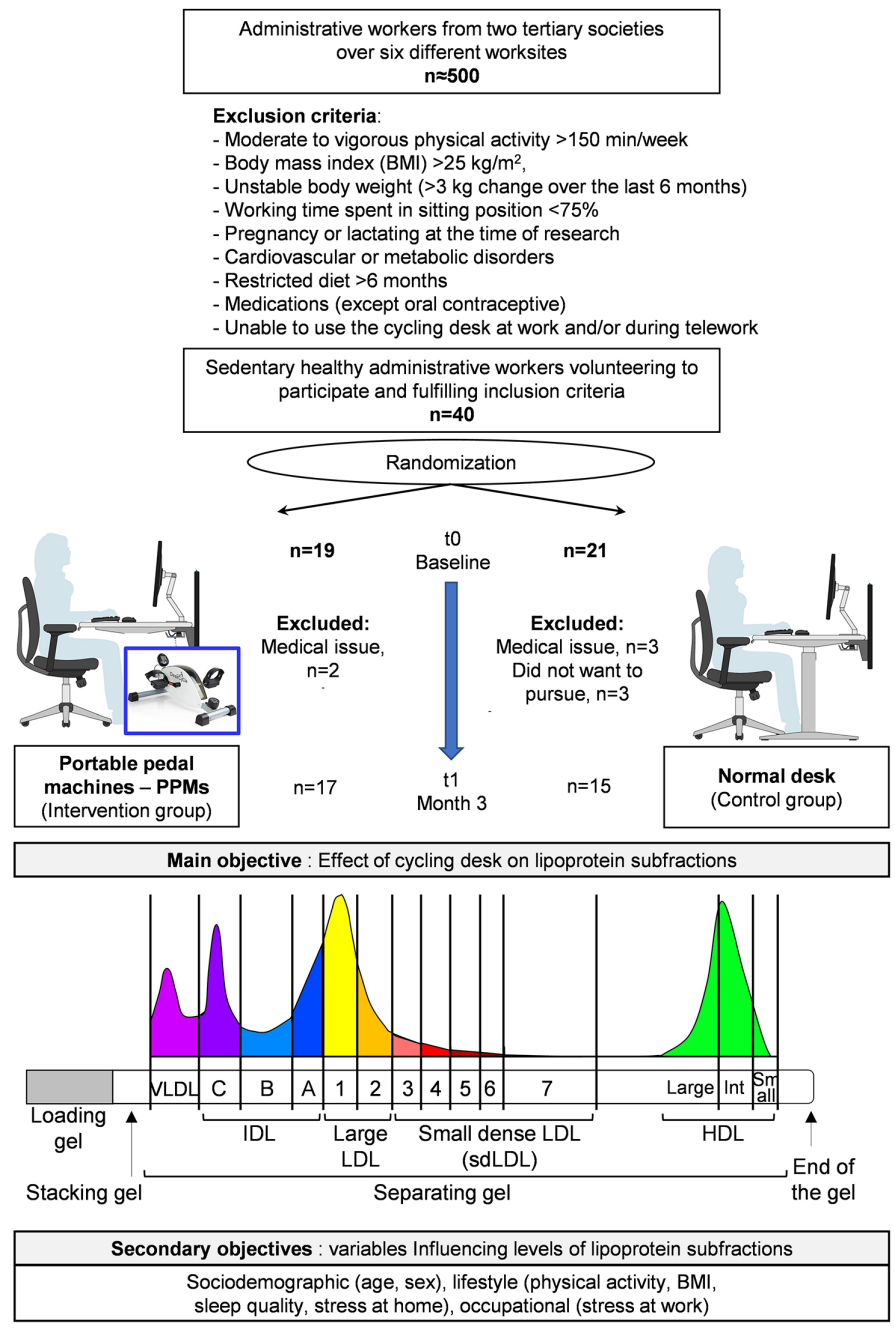
Flow chart and study design

We recruited 40 participants from two tertiary societies in Clermont-Ferrand, France: 19 in the intervention group, and 21 in the control group. All participants were women due to the lack of interest of men to participate in this study. Participants were 43.7 ± 8.6 years old, with a BMI of 23.8 ± 3.4 kg/m^2^; total MVPA was 29.9 ± 15.4 min/day; sedentary at work was 7.7 ± 1.8 h/day; stress at work was 4.49 ± 2.39; stress at home was 2.69 ± 2.17; and sleep quality was 4.88 ± 2.70. There was no difference between groups in any variables at baseline (Table 1). No participants had a metabolic syndrome nor any isolated metabolic abnormality. Five participants (three controls and two from the intervention group) were excluded from the trial due to the medical condition. Three other participants from the control group did not want to participate in t1 measurement. Hence, in this study, 32 participants have finished the trial until t1 (17 in the intervention group and 15 in the control group) (Fig. 1). As instructed, the intervention group cycled on a PPM at work one hour per weekday (59 ± 8 min) for 12 weeks, leading to a significant decrease in sedentary time in the intervention group on the weekdays (from 63.2 to 59.7% of sedentary time) (*p* < 0.05), without changes in the control group (from 66.6 to 64.6% of sedentary time). Even though total MVPA did not differ between groups, there was a significant increase of weekday MPVA in the intervention group (from 22.9 ± 12.1 to 29.3 ± 14.7 min/day, *p* = 0.031) because of PPM use, without changes in the control group (see details in Guirado et al. [20]).

**Table 1:** Baseline comparisons between groups

Characteristics	Intervention group (portable pedal machines - PPMs)	Control group (normal desk)	*p*-value
	*n* = 19	*n* = 21	
Age, years	45.4 ± 8.3	42.3 ± 8.9	0.29
Sex, n female (%)	19 (100%)	21 (100%)	1.00
Body weight, kg	62.7 ± 9.8	61.2 ± 8.7	0.47
Body mass index, kg/m^2^	23.7 ± 3.6	23.8 ± 3.2	0.98
Total Cholesterol, g/l	1.90 ± 0.39	1.81 ± 0.22	0.44
Triglycerides, g/l	0.79 ± 0.33	0.68 ± 0.27	0.39
VLDL, mg/dl	35.6 ± 7.3	34.9 ± 8.2	0.78
Total IDL, mg/dl	42.6 ± 12.1	46.3 ± 9.8	0.46
IDL A, mg/dl	16.1 ± 4.9	18.9 ± 5.7	0.32
IDL B, mg/dl	9.63 ± 2.90	10.2 ± 2.9	0.84
IDL C, mg/dl	16.9 ± 6.0	17.2 ± 5.2	0.74
Total LDL total, mg/dl	95.5 ± 27.8	92.9 ± 14.7	0.78
LDL size, Ångström 10^− 10^ m	271.9 ± 2.5	272.5 ± 1.7	0.47
Large LDL (1–2), mg/dl	50.9 ± 17.5	45.5 ± 10.2	0.15
Small dense LDL [3–7], mg/dl	1.81 ± 1.97	1.23 ± 1.54	0.47
Total HDL, mg/dl	52.2 ± 8.5	49.5 ± 5.8	0.38
Large HDL, mg/dl	21.8 ± 10.8	19.9 ± 7.5	0.90
Intermediate HDL, mg/dl	27.3 ± 5.0	25.4 ± 2.4	0.26
Small HDL, mg/dl	8.6 ± 2.9	9.86 ± 2.32	0.29
MVPA, min/day	30.3 ± 17.7	29.4 ± 12.9	0.78
Sedentary, min/day	502.6 ± 58.0	540.4 ± 74.5	0.14
Stress at work, 0 to 10	4.23 ± 1.89	4.71 ± 2.80	0.54
Stress at home, 0 to 10	2.64 ± 2.10	2.73 ± 2.30	0.92
Sleep quality, 0 to 10	5.30 ± 2.37	4.50 ± 2.98	0.35

Groups did not differ in any parameter at baseline (*p* > 0.10 for each parameter)

### Lipoprotein subfractions

**Fig. 2:**
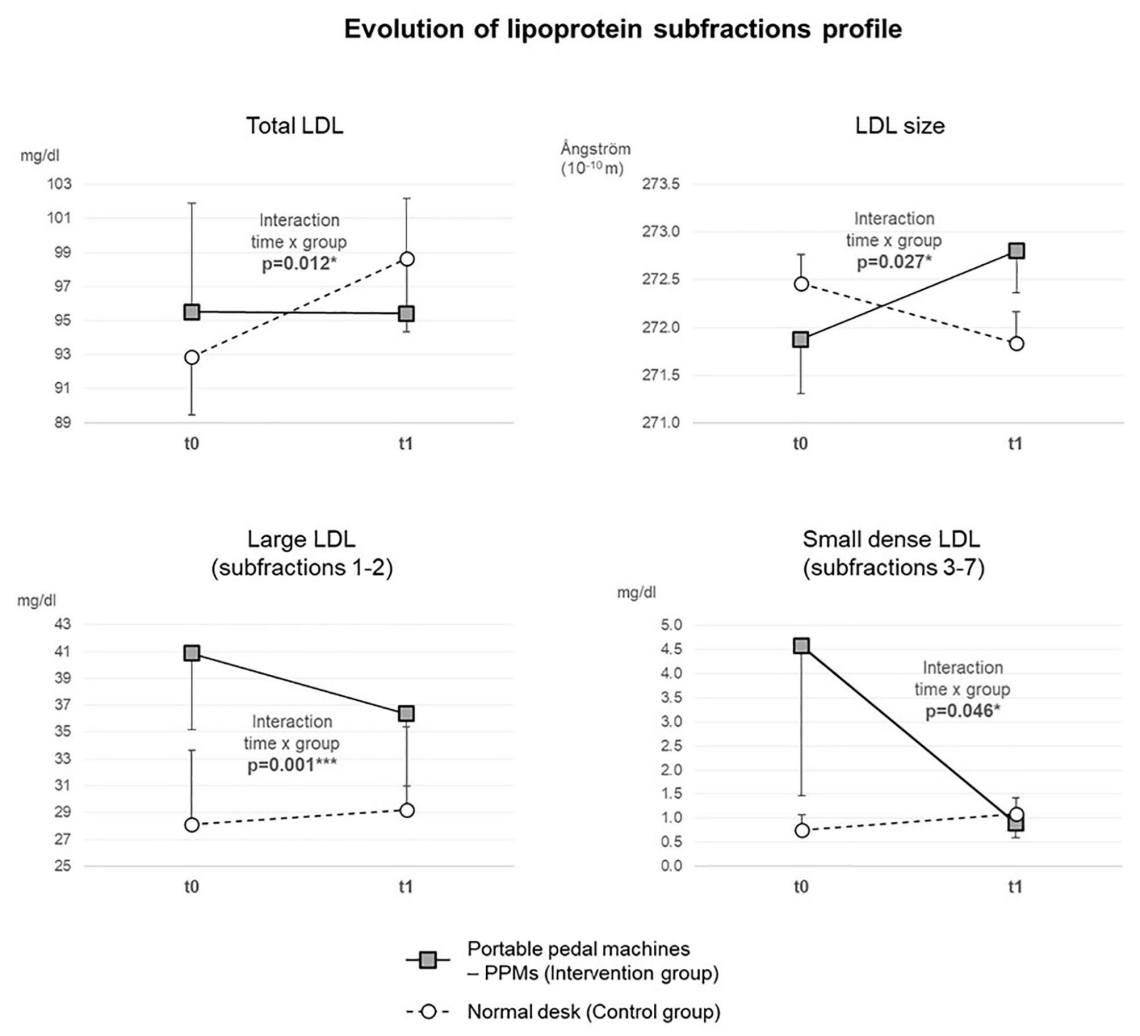
Evolution of lipoprotein subfraction profile t0: Baseline; t1: Month 3; *p ≤ 0.05, **p ≤ 0.01, ***p ≤ 0.001 for interaction time x group from Model 1 without adjustments (for sensitivity analyses, see Table 3)

Changes in lipoprotein subfractions were especially marked for LDL profile. The main results were an interaction time x group for all parameters related to LDL and their subfractions: total LDL-cholesterol (*p* = 0.012), LDL particle size (*p* = 0.027), large LDL subfractions 1 and 2 (*p* = 0.001), and sdLDL subfractions 3 to 7 (*p* = 0.046), using the crude model (all sensitivity analyses were also significant, see below). The interaction reflects difference in the direction of changes between groups. Details of evolution of all subfractions of the lipid profile are available in Table 2. More specifically, total LDL-cholesterol evolved in the control group from 92.9 ± 14.7 at t0 to 98.7 ± 19.0 mg/dl at t1 (*p* = 0.076) and in the intervention group from 95.5 ± 27.8 to 95.4 ± 29.5 mg/dl (*p* = 0.11); the LDL particle size significantly increased in the intervention group from 271.9 ± 2.5 to 272.8 ± 1.9 Ångström (*p* = 0.037) while it did not change in the control group (272.5 ± 1.7 to 271.8 ± 1.5Å (*p* = 0.52); large LDL subfractions 1 and 2 decreased in the intervention group from 50.9 ± 17.5 to 46.1 ± 15.1 mg/dl (*p* = 0.050) while it increased in the control group from 45.5 ± 10.2 to 51.1 ± 10.8 mg/dl (*p* = 0.025); sdLDL subfractions 3 to 7 evolved in the intervention group from 1.81 ± 1.97 to 1.13 ± 1.36 mg/dl (*p* = 0.11) and in the control group from 1.23 ± 1.54 to 1.92 ± 1.44 mg/dl (*p* = 0.59) (Fig. 2). Using paired comparisons, changes between groups differed for total LDL-cholesterol (*p* = 0.007), LDL particle size (*p* = 0.054), and large LDL subfractions 1 and 2 (*p* = 0.005). We did not find any group, time effect, and interaction for triglycerides, VLDL, total IDL and IDL subfractions, as well as HDL subfractions.

**Table 2:** Lipoprotein levels in the intervention group (portable pedal machines – PPMs) and in the control group (normal desk) at baseline (t0) and after 3 months (t1)

	t0	t1	Time effect (intra group comparisons)	Changes between groups	Interaction (time × group)
**Total cholesterol**, g/l					
Intervention (PPMs)	1.90 ± 0.39	1.83 ± 0.39	0.055	**0.014**	0.017**
Controls (normal desk)	1.81 ± 0.22	1.88 ± 0.27	0.12		
**Triglycerides**, g/l					
Intervention (PPMs)	0.79 ± 0.33	0.77 ± 0.31	0.83	0.30	0.57
Controls (normal desk)	0.68 ± 0.27	0.70 ± 0.20	0.53		
**VLDL**, mg/dl					
Intervention (PPMs)	35.6 ± 7.3	36.4 ± 10.4	0.99	0.73	0.66
Controls (normal desk)	34.9 ± 8.1	33.7 ± 6.0	0.99		
**Total IDL**, mg/dl					
Intervention (PPMs)	42.6 ± 12.1	48.0 ± 17.5	0.21	0.99	0.65
Controls (normal desk)	46.3 ± 9.8	45.7 ± 11.4	0.53		
**IDL-A**, mg/dl					
Intervention (PPMs)	16.1 ± 4.9	21.1 ± 8.8	0.08	0.47	0.18
Controls (normal desk)	18.9 ± 5.7	19.2 ± 6.5	0.65		
**IDL-B**, mg/dl					
Intervention (PPMs)	9.63 ± 2.9	10.0 ± 3.6	0.89	0.64	0.99
Controls (normal desk)	10.2 ± 2.9	10.2 ± 3.4	0.87		
**IDL-C**, mg/dl					
Intervention (PPMs)	16.9 ± 5.6	16.9 ± 7.2	0.098	0.13	0.21
Controls (normal desk)	17.2 ± 5.2	16.3 ± 3.6	0.67		
**Total LDL**, mg/dl					
Intervention (PPMs)	95.5 ± 27.8	95.4 ± 29.5	0.11	**0.007**	0.012*
Controls (normal desk)	92.9 ± 14.7	98.7 ± 19.0	0.076		
**LDL size**, Ångström 10^− 10^ m					
Intervention (PPMs)	271.9 ± 2.5	272.8 ± 1.9	0.037*	0.054	0.027*
Controls (normal desk)	272.5 ± 1.7	271.8 ± 1.5	0.52		
**Large LDL** (1–2), mg/dl					
Intervention (PPMs)	50.9 ± 17.5	46.1 ± 15.1	0.050*	**0.005**	0.001***
Controls (normal desk)	45.5 ± 10.2	51.1 ± 10.8	0.025*		
**Small dense LDL** [3–7], mg/dl					
Intervention (PPMs)	1.81 ± 1.97	1.13 ± 1.36	0.11	0.13	0.046*
Controls (normal desk)	1.23 ± 1.54	1.92 ± 1.44	0.59		
**Total HDL**, mg/dl					
Intervention (PPMs)	52.2 ± 8.5	52.5 ± 7.7	0.84	**0.040**	0.07
Controls (normal desk)	49.5 ± 5.8	52.1 ± 6.6	0.041*		
**Large HDL**, mg/dl					
Intervention (PPMs)	21.8 ± 10.8	21.5 ± 7.6	0.75	0.98	0.64
Controls (normal desk)	19.9 ± 7.5	20.3 ± 5.7	0.81		
**Intermediate HDL**, mg/dl					
Intervention (PPMs)	27.3 ± 5.0	27.7 ± 3.9	0.75	0.82	0.42
Controls (normal desk)	25.4 ± 2.4	26.7 ± 2.4	0.28		
**Small HDL**, mg/dl					
Intervention (PPMs)	8.63 ± 2.87	7.76 ± 2.91	0.15	0.60	0.97
Controls (normal desk)	9.86 ± 2.32	9.20 ± 1.66	0.09		

The time effect i.e. intra-group comparisons between t0 and t1 in each group are presented using p-values of paired Student t-test or paired Wilcoxon test if the t-test was not applicable. Between groups comparisons for delta (value at t1 – value at t0) are presented using a Student or Mann-Whitney test if applicable. The interaction (time x group) is presented using the crude model (sensitivity analyses are presented in Table 3). * *p* ≤ 0.05, ** *p* ≤ 0.01, *** *p* ≤ 0.001

**Table 3:** Consistency of *p*-values (significance) for the interaction Time x Group over the different models used for the multivariate analysis (*see details for all lipoprotein subfraction profile in supplementary materials – Supplementary Table**1*)

**Covariables**	**Total LDL**				**LDL size**			
	**Model 1**	**Model 2**	**Model 3**	**Model 4**	**Model 1**	**Model 2**	**Model 3**	**Model 4**
Time effect	0.018*	0.023*	0.042*	0.034*	0.40	0.41	0.56	0.49
Group effect, intervention	0.42	0.48	0.66	0.53	0.45	0.46	0.64	0.67
Interaction Time x Group	0.012*	0.024*	0.044*	0.017*	0.027*	0.029*	0.058	0.068
Age, years	0.72	0.58	0.62	0.86	0.25	0.26	0.27	0.39
BMI, kg/m^2^		0.10	0.16	0.12		0.99	0.92	0.96
MVPA, minutes/day			0.44	0.68			0.38	0.54
Sedentary, minutes/day			0.49	0.31			0.79	0.99
Stress at work, 0 to 10				0.038*				0.49
Stress at home, 0 to 10				0.33				0.36
Sleep quality, 0 to 10				0.44				0.29
	**Large LDL**				**Small dense LDL**			
	**Model 1**	**Model 2**	**Model 3**	**Model 4**	**Model 1**	**Model 2**	**Model 3**	**Model 4**
Time effect	0.013*	0.013*	0.042*	0.06	0.26	0.27	0.48	0.50
Group effect, intervention	0.17	0.20	0.38	0.37	0.40	0.40	0.72	0.73
Interaction Time x Group	0.001***	0.002**	0.007**	0.010**	0.046*	0.048*	0.11	0.12
Age, years	0.97	0.85	0.86	0.97	0.48	0.48	0.49	0.54
BMI, kg/m^2^		**0.049**	0.070	0.079		0.87	0.87	0.94
MVPA, minutes/day			0.70	0.70			0.29	0.32
Sedentary, minutes/day			0.43	0.34			0.45	0.48
Stress at work, 0 to 10				0.60				0.87
Stress at home, 0 to 10				0.94				1.00
Sleep quality, 0 to 10				0.66				0.87

Model 1: crude model; Model 2: crude model plus age, BMI; Model 3: model 2 plus MVPA, sedentary; Model 4: model 3 plus stress at work, stress at home, and sleep quality. *p ≤ 0.05, **p ≤ 0.01, ***p ≤ 0.001

### Sensitivity analyses for the main outcome (lipoprotein subfractions)

Consistency of the results of the interaction time x group was shown in all the four mixed models. For total LDL, there was a consistent significant interaction for all models (*p* < 0.05). For LDL size, the interaction was significant for model 1 (*p* = 0.027) and model 2 (*p* = 0.029), and a tendency close to significance for model 3 (*p* = 0.058) and model 4 (*p* = 0.068). The interaction effect was found on large LDL for all the four models (*p* ≤ 0.01). For sdLDL, the interaction was significant for model 1 (*p* = 0.046) and model 2 (*p* = 0.048), and close to tendency for model 3 (*p* = 0.11) and model 4 (*p* = 0.12). For total HDL, we found interaction effect on model 3 and model 4 (*p* < 0.05) and closer significance for model 1 (*p* = 0.072) and model 2 (*p* = 0.056). In addition, there was a significant interaction for total cholesterol on model 1, model 2, and model 3 (*p* < 0.05). There was no interaction effect for large HDL, VLDL, total IDL, IDL-A, IDL-B, IDL-C, and triglycerides whatever the models.

### Secondary outcomes

**Fig. 3:**
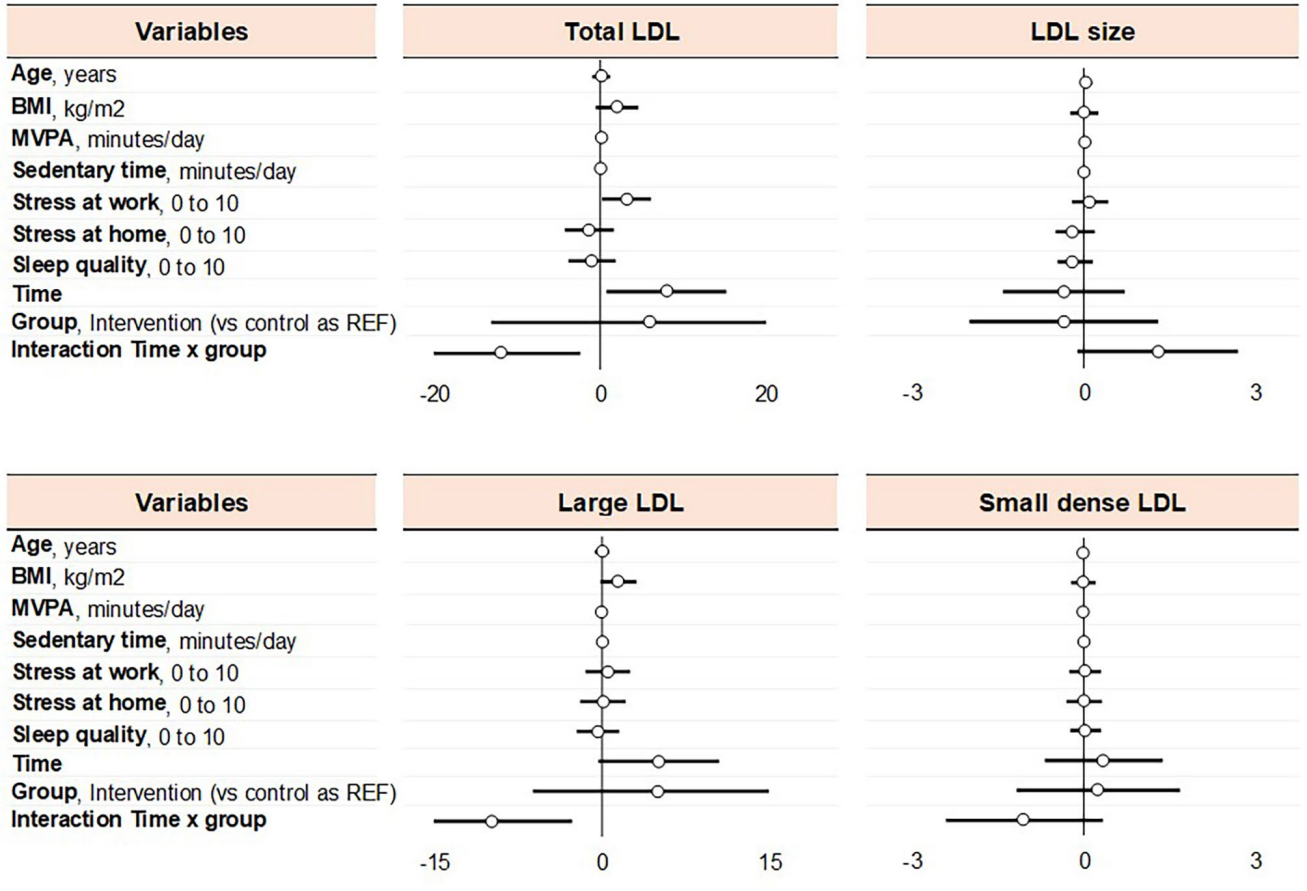
Factors influencing the LDL lipoprotein subfraction profile, using the fully adjusted mixed model (see details for all lipoprotein subfraction profile in supplementary materials – Supplementary Fig. 1)

To assess the factors that can affect the evolution of lipoprotein subfractions, we tested age, BMI, MVPA, sedentary behaviour, stress at work and stress at home, as well as sleep quality, using adjusted mixed models. Coefficients and 95CI are reported below for fully adjusted model (model 4). Total LDL-cholesterol was associated with stress at work: every increase on one point of stress at work is associated with an increase of total LDL of 3.15 mg/dl (95CI 0.20 to 6.11 mg/dl, *p* = 0.038). Large LDL-cholesterol was associated or tended to be associated with BMI in all models (*p* = 0.049 in model 2, *p* = 0.070 in model 3, and *p* = 0.079 in model 4), suggesting that an increase of 1 kg/m^2^ of BMI was associated with an increase of large LDL by 1.42 mg/dl (-0.19 to 3.03 mg/dl) (Fig. 3). A 1 kg/m^2^ increase in BMI was also associated with decreased large HDL by -0.86 mg/dl (-1.70 to -0.01 mg/dl, *p* = 0.047), increased IDL-C by 0.71 mg/dl (0.08 to 1.34 mg/dl *p* = 0.029), and increased triglycerides by 0.04 g/l (0.01 to 0.07 g/l; *p* = 0.003); all models were significant. Aging tended to be associated with an increase in small HDL: each increase of 1 year in age was associated with increased small HDL by 0.09 mg/dl (-0.01 to 0.19 mg/dl; *p* = 0.063); all other models were significant. Lastly, each min/day of sedentary behaviour was associated with a decreased IDL-B by -0.02 mg/dl (-0.03 to -0.003 mg/dl; *p* = 0.021), and an increase of MVPA of 1 min/day was linked with increased in IDL-C by 0.1 mg/dl (0.002 to 0.19; *p* = 0.045) (all models were significant). No variables were found to be associated with VLDL, total IDL, and IDL-A (Supplementary Fig. 1). Eating behaviour also did not influence any of the outcomes.

## Discussion

The main findings were that lipoprotein profile was improved after a 12-week PPMs intervention at work in healthy administrative workers. Changes were mainly showed for total LDL and LDL subfractions. Lipoprotein profile was worsened by stress at work, BMI and age.

### Effects of PPMs at work on lipoprotein subfraction profile

Our main findings were that 60 min of light intensity physical activity at work using PPMs was associated with improvement of lipoprotein subfraction profile, particularly a decrease on total LDL, large LDL, and sdLDL. This improvement, otherwise, was not found in the control group. To our knowledge, this is the first study to investigate the effect of occupational PPMs intervention on lipoprotein subfractions. In the intervention group, the increase in LDL particle size indicated the shift of LDL subfractions from sdLDL towards larger LDL particles [10, 24]. Large LDL also decreased in the intervention group. Interestingly, the role of Large LDL in the atherosclerotic process is still under debate. While some studies traditionally viewed Large LDL as less atherogenic [11, 33], some studies associated Large LDL as an independent predictor of myocardial infarction, as highly atherogenic, and responsible for premature atherosclerosis [34]. Large LDL could be viewed as harmful as sdLDL [10]. Despite short duration of the treatment, the changes on sdLDL can be related to the effect of light-intensity physical activity [20, 24]. While much research reported the benefit of leisure MVPA on lipoprotein levels [35–38], the effectiveness of leisure light physical exercise to reduce the level of LDL remains debatable. Although some studies did not show any effect of leisure light aerobic exercise on lipoprotein subfraction profile in sedentary healthy individuals [39, 40], sdLDL and LDL size improved in patient with hypercholesterolemia [41]. We did not find an influence of PPMs on IDL, VLDL, and HDL subfractions. Interestingly, the improvement of lipoprotein profile by light physical activity has been suggested to be mediated by tissue-specific effects on lipoprotein lipase (LPL); those effects were reported for HDL, LDL, VLDL, and triglycerides metabolism [42, 43].

### Influencing variables of lipoprotein subfraction profile

We identified age as a factor increasing small HDL, in line with some studies that reported an increased cardiovascular risk with age, possibly through changes in lipoprotein profile [22, 44, 45]. Although our data did not permit to analyse the effect of sex on lipoprotein profile, literature is inconsistent. For example, higher sedentary behaviour in women, but not in men, was associated with increased sdLDL and decreased large HDL i.e. higher cardiovascular risk [23], while some studies did not show any sex effect [46, 47]. In our study, higher BMI was associated with decreased large HDL, but also increased large LDL, IDL-C, and triglycerides. Traditionally, higher BMI was associated with cardiovascular risk and atherogenic lipoproteins profile [37, 48–50], which is in line with our results, except for the relation BMI / Large LDL that should be a protective factor. It may be partially explained because participants were all women, not obese, and mostly premenopausal. Middle age is associated with significant changes in body composition that could be not necessarily deleterious [51, 52].We found that MVPA and sedentary behaviour were associated with increased IDL-C and IDL-B, respectively. Although these relations were not found in the literature, the importance of MVPA to improve the lipoprotein subfraction profile has been largely studied as well as the effects of sedentary behaviour. Most interventional studies on lipoprotein profile combined physical activity and diet in obese individuals, demonstrating reduced small HDL subfractions [10] or sdLDL particles [53]. Studies on the effects on MVPA in non-obese normolipidemic volunteers are less common but exist, also moving the lipoprotein profile from smaller to larger LDL – classically less atherogenic [24]. Sedentary behaviour was previously associated with higher sdLDL and lower large LDL [54]. The increasing sedentary behaviour in workers can trigger the development of coronary artery disease [55, 56], and must be considered as an occupational risk [18]. Promoting adjustable table, PPMs or cycling desk in the workplace is aiming to reduce sedentary behaviour and to prevent chronic diseases [48, 49, 57], and we report the first data on lipoprotein subfraction profile. Interestingly, we also found occupational stress being associated with total LDL. From the INTER-HEART study, stress at work is recognized a major cardiovascular risk [25]. Stress at work contributes to unfavourable lipoprotein profile [27, 28], that can be explained by stress-induced hormonal changes, such as increased cortisol levels that affect lipoprotein metabolism [50, 58]. Lastly, even if we did not find any association between sleep quality and lipoprotein subfractions, better sleep quality was traditionally associated with a better lipid profile and thus lower cardiovascular risk [59, 60].

### Limitations

Our study has some limitations. First, our study has a limited sample size. However, this study is very novel as it is the first study assessing the effects of active workstation on lipoprotein subfraction profile in healthy sedentary workers. The limited small sample size was partly due to recruitment difficulties linked with the Covid-19 pandemic when the study was conducted, which may be another source of bias [61, 62]. Nevertheless, the study was a randomized controlled trial, precluding recruitment bias as well as the bias related with external factors. Still, the sample size limited the generalisability of our results. Despite not intentional, we only recruited women. The absence of male participants was also a factor precluding generalisability of our results, but it was also a factor limiting putative sex bias – as the lipoprotein subfraction profile is influenced by sex. Some variables were not measured in our intervention study, such as deeper biomolecular pathways that could have explained the observed changes in lipoprotein profile [63, 64]. Selection bias in our study may have occurred. As our recruitment was voluntary-based, we could not anticipate whether volunteers may have been the most sedentary workers, or those who already wanted to change their behaviour [65]. Some data were self-reported, such as stress at work and stress at home, as well as sleep quality, but those scales are commonly used in the literature, and there is no other way to measure subjective feelings [66]. However, our main outcome was the lipoprotein subfractions measured by Lipoprint® electrophoresis, i.e. an objective biological parameter [67] – and we also measured all lipoprotein subfractions. Further studies should be conducted with a larger sample size involving more companies on several geographical sites for better population representativeness. Future studies should also investigate more deeply on behaviours, diet, and work characteristics.

## Conclusion

Lipoprotein subfraction profile was improved after 12-weeks of PPMs use in the office at work in healthy sedentary administrative workers. Changes were mainly shown for LDL and LDL subfractions (LDL size, large LDL, and small dense LDL). Besides the influence of BMI on lipoprotein subfractions, stress at work also seemed to worsen the lipoprotein profile. Despite this study was a randomized controlled trial, further studies should keep the same design but with a bigger sample size, a prolonged follow-up, and involving both men and women, with additional biological measures of lipid metabolism.

### Electronic supplementary material

Below is the link to the electronic supplementary material.

Supplementary Material 1

## Data Availability

No datasets were generated or analysed during the current study.

## Abbreviations

BMI: Body max index
CHU: Centre hospitalier universitaire
DEXA: Dual x-ray absorptiometry
HDL: High-density lipoprotein
HDL-C: High-density lipoprotein cholesterol
IDL: Intermediate-density lipoprotein
IDL-C: Intermediate-density lipoprotein cholesterol
LDL: Low-density lipoprotein
LDL-C: Low density lipoprotein cholesterol
LPL: Lipoprotein lipase
MVPA: Moderate-to-vigorous physical activity
PA: Physical activity
PPMs: Portable pedal machines
SB: Sedentary behaviour
sdLDL: Small dense low density lipoprotein
TG: Triglyceride
VLDL: Very-low-density lipoprotein
