# Supplementary material for: Effects of portable pedal machines at work on lipoprotein subfraction profile in sedentary workers – the REMOVE study
Source: Lipids Health Dis. 2024 Apr 14;23:105. doi: 10.1186/s12944-024-02098-w (PMC11016206; doi:10.1186/s12944-024-02098-w)
Supplement: Supplementary file 1 — Supplementary Material 1 [file 12944_2024_2098_MOESM1_ESM.docx]

**Supplementary materials**

**Supplementary Table 1**. Consistency of p-values (significance) for the interaction Time x Group over the different models used for the multivariate analysis.

*Model 1: crude model; Model 2: crude model plus age, BMI; Model 3: model 2 plus MVPA, sedentary; Model 4: model 3 plus stress at work, stress at home, and sleep quality. *p≤.05, **p≤.01, ***p≤.001*

|  |  | **Total Cholesterol** | | | |  | **Triglycerides** | | | |
| --- | --- | --- | --- | --- | --- | --- | --- | --- | --- | --- |
|  |  | **Model 1** | **Model 2** | **Model 3** | **Model 4** |  | **Model 1** | **Model 2** | **Model 3** | **Model 4** |
| Time effect |  | 0.070 | 0.081 | 0.14 | 0.29 |  | 0.60 | 0.67 | 0.71 | 0.99 |
| Group effect, intervention |  | 0.49 | 0.53 | 0.63 | 0.64 |  | 0.30 | 0.35 | 0.39 | 0.38 |
| Interaction Time x Group |  | **0.017*** | **0.026*** | **0.041*** | 0.095 |  | 0.57 | 0.75 | 0.76 | 0.94 |
| Age, years |  | 0.57 | 0.46 | 0.49 | 0.69 |  | 0.82 | 0.87 | 0.89 | 0.71 |
| BMI, kg/m^2^ |  |  | 0.21 | 0.25 | 0.23 |  |  | **0.004**** | **0.005**** | **0.003**** |
| MVPA, minutes/day |  |  |  | 0.52 | 0.50 |  |  |  | 0.56 | 0.31 |
| Sedentary, minutes/day |  |  |  | 0.54 | 0.36 |  |  |  | 0.86 | 0.63 |
| Stress at work, 0 to 10 |  |  |  |  | 0.86 |  |  |  |  | 0.90 |
| Stress at home, 0 to 10 |  |  |  |  | 0.48 |  |  |  |  | 0.054 |
| Sleep quality, 0 to 10 |  |  |  |  | 0.55 |  |  |  |  | 0.75 |

|  |  | **VLDL** | | | |
| --- | --- | --- | --- | --- | --- |
|  |  | **Model 1** | **Model 2** | **Model 3** | **Model 4** |
| Time effect |  | 0.65 | 0.65 | 0.39 | 0.38 |
| Group effect, intervention |  | 0.86 | 0.97 | 0.80 | 0.89 |
| Interaction Time x Group |  | 0.66 | 0.55 | 0.33 | 0.36 |
| Age, years |  | 0.12 | 0.069 | 0.11 | 0.20 |
| BMI, kg/m^2^ |  |  | 0.056 | 0.12 | 0.097 |
| MVPA, minutes/day |  |  |  | 0.88 | 0.94 |
| Sedentary, minutes/day |  |  |  | 0.090 | 0.092 |
| Stress at work, 0 to 10 |  |  |  |  | 0.32 |
| Stress at home, 0 to 10 |  |  |  |  | 0.89 |
| Sleep quality, 0 to 10 |  |  |  |  | 0.86 |

|  |  | **Total IDL** | | | |  | **IDL-A** | | | |
| --- | --- | --- | --- | --- | --- | --- | --- | --- | --- | --- |
|  |  | **Model 1** | **Model 2** | **Model 3** | **Model 4** |  | **Model 1** | **Model 2** | **Model 3** | **Model 4** |
| Time effect |  | 0.66 | 0.66 | 0.62 | 0.75 |  | 0.68 | 0.67 | 0.65 | 0.93 |
| Group effect, intervention |  | 0.77 | 0.68 | 0.64 | 0.75 |  | 0.31 | 0.30 | **0.346** | **0.353** |
| Interaction Time x Group |  | 0.65 | 0.57 | 0.63 | 0.68 |  | 0.18 | 0.18 | 0.21 | 0.18 |
| Age, years |  | 0.40 | 0.31 | 0.39 | 0.68 |  | 0.15 | **0.142** | **0.181** | 0.54 |
| BMI, kg/m^2^ |  |  | 0.23 | 0.36 | 0.21 |  |  | 0.66 | 0.79 | 0.52 |
| MVPA, minutes/day |  |  |  | 0.21 | 0.32 |  |  |  | 0.74 | 0.92 |
| Sedentary, minutes/day |  |  |  | 0.58 | 0.34 |  |  |  | 0.95 | 0.70 |
| Stress at work, 0 to 10 |  |  |  |  | 0.094 |  |  |  |  | 0.56 |
| Stress at home, 0 to 10 |  |  |  |  | 0.80 |  |  |  |  | 0.44 |
| Sleep quality, 0 to 10 |  |  |  |  | 0.45 |  |  |  |  | 0.086 |

|  |  | **IDL-B** | | | |  | **IDL-C** | | | |
| --- | --- | --- | --- | --- | --- | --- | --- | --- | --- | --- |
|  |  | **Model 1** | **Model 2** | **Model 3** | **Model 4** |  | **Model 1** | **Model 2** | **Model 3** | **Model 4** |
| Time effect |  | 0.88 | 0.86 | 0.78 | 0.66 |  | 0.83 | 0.87 | 0.73 | 0.77 |
| Group effect, intervention |  | 0.77 | 0.69 | 0.26 | 0.29 |  | 0.63 | 0.74 | 0.81 | 0.72 |
| Interaction Time x Group |  | 0.98 | 0.93 | 0.57 | 0.58 |  | 0.21 | 0.30 | 0.21 | 0.15 |
| Age, years |  | 0.24 | 0.17 | 0.22 | 0.46 |  | 0.88 | 0.91 | 0.99 | 0.88 |
| BMI, kg/m^2^ |  |  | 0.21 | 0.30 | 0.14 |  |  | **0.023*** | **0.034*** | **0.029*** |
| MVPA, minutes/day |  |  |  | 0.54 | 0.66 |  |  |  | **0.018*** | **0.045*** |
| Sedentary, minutes/day |  |  |  | **0.040*** | **0.021*** |  |  |  | 0.26 | 0.18 |
| Stress at work, 0 to 10 |  |  |  |  | 0.18 |  |  |  |  | 0.11 |
| Stress at home, 0 to 10 |  |  |  |  | 0.85 |  |  |  |  | 0.28 |
| Sleep quality, 0 to 10 |  |  |  |  | 0.48 |  |  |  |  | 0.45 |

| **Covariables** |  | **Total LDL** | | | |  | **LDL size** | | | |
| --- | --- | --- | --- | --- | --- | --- | --- | --- | --- | --- |
|  |  | **Model 1** | **Model 2** | **Model 3** | **Model 4** |  | **Model 1** | **Model 2** | **Model 3** | **Model 4** |
| Time effect |  | **0.018*** | **0.023*** | **0.042*** | **0.034*** |  | 0.40 | 0.41 | 0.56 | 0.49 |
| Group effect, intervention |  | 0.42 | 0.48 | 0.66 | 0.53 |  | 0.45 | 0.46 | 0.64 | 0.67 |
| Interaction Time x Group |  | **0.012*** | **0.024*** | **0.044*** | **0.017*** |  | **0.027*** | **0.029*** | 0.058 | 0.068 |
| Age, years |  | 0.72 | 0.58 | 0.62 | 0.86 |  | 0.25 | 0.26 | 0.27 | 0.39 |
| BMI, kg/m^2^ |  |  | 0.10 | 0.16 | 0.12 |  |  | 0.99 | 0.92 | 0.96 |
| MVPA, minutes/day |  |  |  | 0.44 | 0.68 |  |  |  | 0.38 | 0.54 |
| Sedentary, minutes/day |  |  |  | 0.49 | 0.31 |  |  |  | 0.79 | 0.99 |
| Stress at work, 0 to 10 |  |  |  |  | **0.038*** |  |  |  |  | 0.49 |
| Stress at home, 0 to 10 |  |  |  |  | 0.33 |  |  |  |  | 0.36 |
| Sleep quality, 0 to 10 |  |  |  |  | 0.44 |  |  |  |  | 0.29 |

|  |  | **Large LDL** | | | |  | **Small dense LDL** | | | |
| --- | --- | --- | --- | --- | --- | --- | --- | --- | --- | --- |
|  |  | **Model 1** | **Model 2** | **Model 3** | **Model 4** |  | **Model 1** | **Model 2** | **Model 3** | **Model 4** |
| Time effect |  | **0.013*** | **0.013*** | **0.042*** | 0.06 |  | 0.26 | 0.27 | 0.48 | 0.50 |
| Group effect, intervention |  | 0.17 | 0.20 | 0.38 | 0.37 |  | 0.40 | 0.40 | 0.72 | 0.73 |
| Interaction Time x Group |  | **0.001***** | **0.002**** | **0.007**** | **0.010**** |  | **0.046*** | **0.048*** | 0.11 | 0.12 |
| Age, years |  | 0.97 | 0.85 | 0.86 | 0.97 |  | 0.48 | 0.48 | 0.49 | 0.54 |
| BMI, kg/m^2^ |  |  | **0.049** | 0.070 | 0.079 |  |  | 0.87 | 0.87 | 0.94 |
| MVPA, minutes/day |  |  |  | 0.70 | 0.70 |  |  |  | 0.29 | 0.32 |
| Sedentary, minutes/day |  |  |  | 0.43 | 0.34 |  |  |  | 0.45 | 0.48 |
| Stress at work, 0 to 10 |  |  |  |  | 0.60 |  |  |  |  | 0.87 |
| Stress at home, 0 to 10 |  |  |  |  | 0.94 |  |  |  |  | 1.00 |
| Sleep quality, 0 to 10 |  |  |  |  | 0.66 |  |  |  |  | 0.87 |

|  |  | **Total HDL** | | | |  | **Large HDL** | | | |
| --- | --- | --- | --- | --- | --- | --- | --- | --- | --- | --- |
|  |  | **Model 1** | **Model 2** | **Model 3** | **Model 4** |  | **Model 1** | **Model 2** | **Model 3** | **Model 4** |
| Time effect |  | 0.081 | 0.082 | **0.050** | 0.090 |  | 0.92 | 0.95 | 0.91 | 0.95 |
| Group effect, intervention |  | 0.17 | 0.13 | 0.097 | 0.052 |  | 0.52 | 0.34 | 0.28 | 0.27 |
| Interaction Time x Group |  | 0.072 | 0.056 | **0.029*** | **0.031*** |  | 0.63 | 0.47 | 0.37 | 0.39 |
| Age, years |  | 0.89 | 0.93 | 0.99 | 0.50 |  | 0.56 | 0.29 | 0.35 | 0.24 |
| BMI, kg/m^2^ |  |  | 0.073 | 0.21 | 0.45 |  |  | **0.008**** | **0.040*** | **0.047*** |
| MVPA, minutes/day |  |  |  | 0.22 | 0.27 |  |  |  | 0.58 | 0.67 |
| Sedentary, minutes/day |  |  |  | 0.80 | 0.81 |  |  |  | 0.55 | 0.81 |
| Stress at work, 0 to 10 |  |  |  |  | 0.15 |  |  |  |  | 0.62 |
| Stress at home, 0 to 10 |  |  |  |  | 0.25 |  |  |  |  | 0.80 |
| Sleep quality, 0 to 10 |  |  |  |  | 0.57 |  |  |  |  | 0.22 |

|  |  | **Intermediate HDL** | | | |  | **Small HDL** | | | |
| --- | --- | --- | --- | --- | --- | --- | --- | --- | --- | --- |
|  |  | **Model 1** | **Model 2** | **Model 3** | **Model 4** |  | **Model 1** | **Model 2** | **Model 3** | **Model 4** |
| Time effect |  | 0.24 | 0.24 | 0.15 | 0.26 |  | 0.29 | 0.20 | 0.10 | 0.087 |
| Group effect, intervention |  | 0.23 | 0.18 | 0.056 | **0.036*** |  | 0.058 | 0.057 | **0.033*** | **0.034*** |
| Interaction Time x Group |  | 0.44 | 0.37 | 0.21 | 0.31 |  | 0.95 | 0.93 | 0.70 | 0.56 |
| Age, years |  | 0.22 | 0.31 | 0.21 | 0.45 |  | **0.032*** | **0.032*** | **0.039*** | 0.063 |
| BMI, kg/m^2^ |  |  | 0.16 | 0.48 | 0.99 |  |  | 0.72 | 0.74 | 0.62 |
| MVPA, minutes/day |  |  |  | 0.99 | 0.95 |  |  |  | 0.45 | 0.31 |
| Sedentary, minutes/day |  |  |  | 0.20 | 0.24 |  |  |  | 0.090 | 0.13 |
| Stress at work, 0 to 10 |  |  |  |  | 0.27 |  |  |  |  | 0.69 |
| Stress at home, 0 to 10 |  |  |  |  | 0.17 |  |  |  |  | 0.16 |
| Sleep quality, 0 to 10 |  |  |  |  | 0.46 |  |  |  |  | 0.25 |

**Supplementary Figure 1**. Factors influencing the lipoprotein subfraction profile, using the fully adjusted mixed model.

*The effect of each variable on the level of lipoprotein subfractions is represented by a dot on a horizontal line in the forest-plot. The dots represent the coefficient for each variable, and the length of each line around the dots represent their 95% confidence interval (95CI). The black solid vertical line represents the null estimate (with a value of 0). Horizontal lines that cross the null vertical line represent non-significant variables on the levels of each lipoprotein subfractions.*
